# Effect of Neonatal Interventions with Specific Micronutrients and Bovine Colostrum on Micronutrient and Oxidative Statuses and on Gut Microbiota in Piglets from Birth to Post-Weaning Period

**DOI:** 10.3390/vetsci12020151

**Published:** 2025-02-10

**Authors:** Lucie Galiot, Isabelle Audet, Bazoumana Ouattara, Nathalie Bissonnette, Guylaine Talbot, Frédéric Raymond, Thomas Deschênes, Martin Lessard, Jérôme Lapointe, Frédéric Guay, Jean Jacques Matte

**Affiliations:** 1 Département des Sciences Animales, Université Laval, Ville de Québec, QC G1V 0A6, Canada; lgaliot@cdpq.ca (L.G.); frederic.guay@fsaa.ulaval.ca (F.G.); 2 Agriculture et Agroalimentaire Canada, Centre de Recherche et de Développement de Sherbrooke, Sherbrooke, QC J1M 0C8, Canada; isabelle.audet@agr.gc.ca (I.A.); bazouma.ouattara@upgc.edu.ci (B.O.); nathalie.bissonnette@agr.gc.ca (N.B.); guylaine.talbot@agr.gc.ca (G.T.); mlessard0905@gmail.com (M.L.); jerome.lapointe@agr.gc.ca (J.L.); 3 Biological Sciences, Animal Biology, Université Peleforo GON COULIBALY, Korhogo 1328, Côte d’Ivoire; 4 École de Nutrition, Centre Nutrition, Santé et Société (NUTRISS), et Institut de la Nutrition et des Aliments Fonctionnels (INAF), Université Laval, Ville de Québec, QC G1V 0A6, Canada; frederic.raymond@fsaa.ulaval.ca (F.R.); thomas.deschenes@inrs.ca (T.D.)

**Keywords:** micronutrients, weaning, microbiota, antioxidants, piglets

## Abstract

This study aimed to determine the impact of supplementations of copper, vitamins A and D (ADCU), and a bovine colostrum extract (BC) on the micronutrient status, antioxidant status, and intestinal microbiota of piglets until the post-weaning period. Twenty-three sows were fed conventional gestation and lactation diets, and twenty-four sows were fed conventional diets supplemented with ADCU. For each litter, all piglets received one of four treatments during lactation: no supplementation; ADCU; BC; and ADCU + BC. Within each litter, one low (LW) and one high birth weight (HW) piglet were euthanized before and after weaning to collect liver and intestinal samples. Serum vitamin D, liver retinol, and liver Cu were greater in ADCU piglets (*p* < 0.01), mostly before weaning. After weaning, liver Cu decreased markedly with a drop of 75% in all treatments, despite high levels of Cu in their post-weaning diets. The antioxidant status of piglets was not globally altered by treatments (*p* > 0.05). For microbiota composition, sow supplementation increased (*p* < 0.01) richness in bacterial species in the piglet colon, either before or shortly after weaning. Short-chain fatty acids in caecal digesta were increased by sow supplementation in LW piglets before weaning at 16 days of age (*p* < 0.05). In conclusion, oral supplementations to piglets increased postnatal micronutrient statuses during lactation, but this did not generally persist after weaning. Treatments to sows or piglets did not improve the response of piglets to oxidative stress, but supplementation to sows favoured gut microbiota diversity, particularly in LW piglets.

## Simple Summary

Placental and colostral transfers of copper and vitamins A and D, key micronutrients for antioxidant metabolism and the development of immunity and intestinal microbiota, are limited in pig species. Hence, supplementing the three above-mentioned micronutrients indirectly through sow’s diets or directly to suckling piglets along with a bovine colostrum extract might contribute to the robustness and health of piglets and eventually to their growth performance during the pre- and post-weaning period. The present results indicated that direct supplementations of micronutrients to piglets with or without a bovine colostrum extract, though efficient in transiently increasing the postnatal micronutrient status during lactation, had no apparent impact on responses to oxidative stress or gut microbiota in suckling and post-weaned piglets. Similar responses were observed from supplementations through the sow’s diets in late gestation and lactation except for gut microbiota, where apparent beneficial impacts were observed, particularly in suckling, low birth weight piglets. Taken together, the present results indicated that the stress of weaning is a crucial factor in treatments or birth weight class effects for the metabolic status of these micronutrients, the antioxidative status, or the microbiota of piglets. Further studies will be needed to investigate the impact of supplementations administered closer to the onset of weaning stress on the robustness and health of piglets.

## 1. Introduction

Hyperprolificity in sow populations has increased productivity in the last decades, but this was achieved at the expense of other critical traits such as homogeneity of birth weights within litter [1,2]. Low-weight (LW) piglets consume less colostrum than high-weight (HW) littermates [3], which may trigger an inadequacy of micronutrients transfer for the postnatal growth and health of these animals [4,5]. Ultimately, LW piglets develop, within the first three weeks of life, an altered immune response, markedly associated with activation of inflammatory pathways [6] and deleterious effects on oxidative–antioxidative balance [7], and this is concomitant with slower growth and less chances of survival as compared with HW piglets [8]. Moreover, the colonization of intestinal microbiota during lactation also differs between littermates, with more beneficial bacteria in HW piglets than in LW piglets [9], with consequences on short-chain fatty acids (SCFAs) such as acetic, butyric, and propionic acid being produced from bacteria and being recognized as beneficial metabolites for piglet intestinal health [10]. These differences between littermates are even more critical because weaning is a stressful period for all piglets with major dysfunctions of intestinal and immune systems and gut microbiota dysbiosis, resulting in reduced health, feed intake, and overall performances [11,12,13].

Placental and colostral transfers of copper (Cu) and vitamins A and D are limited in piglets [14]. These micronutrients are recognized as key elements for antioxidant metabolism and the development of immunity and intestinal microbiota [4,15,16,17]. Besides its micronutrient contents, the colostrum also provides growth factors [18] that may improve the development of immune competence [6], development of intestinal barrier function, and establishment of beneficial bacteria in the microbiota in piglets [5,19].

Therefore, it was hypothesized that supplementing the three above-mentioned micronutrients and/or a bovine colostrum extract (BC) during the suckling period might contribute to the robustness and health of suckling piglets, particularly for LW individuals, up to and during the peri-weaning period. The present study aimed to assess the impact of providing a supplemental provision of Cu and vitamins A and D indirectly through sow diets and/or directly to suckling piglets with or without BC on micronutrient metabolism, responses to oxidative status, and establishment of gut microflora during lactation and after weaning, according to birth weight classes of these young animals. The present study was complementary to two others, one [20] using similar treatments and different indicators within the limitations of commercial conditions, and another one aligned on the development of immune competence in piglets [6].

## 2. Material and Methods

### 2.1. Animal Management and Data Collection

Nulliparous sows (Yorkshire × Landrace, Groupe Ceres Inc., Saint-Nicolas, QC, Canada) were inseminated with Duroc semen to obtain 47 pregnant sows with litters of at least 10 piglets. They were fed gestation and lactation diets as described by Galiot et al. [21] (Supplementary Table S1). Feed intake was limited to 2.7 kg per day until day 110 of gestation and 3.7 kg per day until farrowing. The sows were provided ad libitum access to feed during lactation. One group of 23 sows was fed conventional diets (CONT), and one group of 24 sows received CONT diets with extra daily supplements of 25-hydroxycholecalciferol (4 ĸIU, DSM Nutritional Products, Ayr, ON, Canada), β-carotene (30 ĸIU, DSM Nutritional Products), and Cu proteinate (45 mg, Alltech, Guelph, ON, Canada) from 90 days of gestation to parturition (SUPPL). This dietary supplementation was pursued and doubled during lactation. Litter sizes were standardized within 24 h after farrowing to a minimum of 10 and maximum of 12 piglets per sow, and adoptions only occurred within the same treatment group. For each group of sows, all piglets within each litter were assigned to one of the following treatments: (CON) control without other interventions than usual practice; (ADCU) oral administration of retinol acetate (DSM Nutritional Product) at 2 (25 ĸIU) and 8 days (50 ĸIU) of age, 25-hydroxycholecalciferol (4 ĸIU and 8 ĸIU, respectively, DSM Nutritional Product, Ayr, ON, Canada) and Cu proteinate (4 and 8 mg, respectively, Alltech, St-Hyacinthe, QC, Canada) with exposure to ultraviolet B (UVB) lights (30 min per day every other day) from 5 to 19 days of age; (BC) oral administration of BC given daily (4 g, Saskatoon Colostrum Company Ltd., Saskatoon, SK, Canada, diluted in 10 mL of water) from 5 to 10 days of age; and (ADCU + BC) combination of BC and ADCU treatments. Routes, modes, and levels of administration of Cu, vitamin A, and vitamin D to sows and piglets were based on a previous study [21]. The total amounts of three micronutrients brought by the BC extract were negligible with an estimated contribution (based on analytical values) of 24 IU, 0 IU, and 0.004 mg per day for vitamin A, vitamin D, and Cu, respectively. Regarding the vitamin D source for ADCU treatment, the whole litter was moved to a small pen equipped with a 40-watt UVB lamp positioned 1.2 m above the ground, as described by Cooper et al. [22]. They were exposed to this radiation for 30 min every two days from 3 to 19 days of age.

For all sows, blood samples were collected at 89 days of gestation (before initiation of treatments), at 110 days of gestation, and at weaning by jugular vein puncture with trace element-free Vacutainer^®^ tubes (Vacutainer, Becton Dickinson, Franklin Lakes, NJ, USA) to measure the concentrations of retinol, 25-hydroxycholecalciferol (25-OH-D_3_), and copper in blood serum. Body weights of sows were also recorded at 89 and 110 days of gestation and 1 day after weaning (21 days of lactation). Milk samples were collected from sows at 2 weeks of lactation following injection of oxytocin (i.v., 20 IU) for the determination of micronutrients in milk. Within each litter, piglets were divided into three classes based on their weight at 1 day of age: 4 low (LW)-, 4 high (HW)-, and 4 medium-weight (MW) piglets. Global average body weights per class were 1.19 ± 0.1, 1.48 ± 0.01, and 1.75 ± 0.01 kg for LW, MW, and HW, respectively. Growth performance indicators were monitored for MW piglets at 1, 8, 21 (average daily gain), 28, 35, and 42 days of age (average daily gain, feed intake, and gain to feed ratio). Teeth filing, tail clipping, and ear tagging were performed one day after birth, and iron (1 mL of iron dextran containing 100 mg Fe/mL, Ironol, P.V.U., Lavaltrie, QC, Canada) was injected at 3 days of age for all piglets. No creep feed was offered during lactation. For all litters, blood samples were collected from MW piglets at 2 and 8 days of age (prior to administration of treatments) as well as at 21 (weaning) and 42 days of age for the determination of micronutrients in plasma. After weaning, LW, HW, and MW littermates were each moved into three different pens until the end of the experiment at 42 days of age. An individual feeder was allocated to each pen, and pigs had ad libitum access to water and to the same feed. Two different types of commercial diets were given to all piglets after weaning: the first was given from 21 to 28 days of age and the second from 28 to 42 days of age. Diets contained (analytical values), respectively, 18.6 and 19.5% of CP; 6.0 and 4.7% of fat; 2.2 and 2.7% of crude fiber; 124 and 111 mg/kg of Cu; 7000 and 9000 IU of vitamin A; and 2300 and 1900 IU of vitamin D, respectively. A pair of piglets (1 LW and 1 HW) from each litter was selected and sacrificed at 16, 23, and 42 days of age for collections of blood, liver and intestine samples, as well as collection of caecal and colon content. These samples were immediately frozen and stored at −80 °C. For intestinal samples, the tissue was excised immediately after death, and the jejunum were separated from the rest of the intestinal tract. Portions of 10 cm from the mid-jejunum were collected, opened longitudinally, and washed in phosphate-buffered saline. The mucosa was then collected by scraping with clean glass slides and immediately frozen in liquid nitrogen and stored at −80 °C until assays were performed. For the microbiota, only the colon content was used.

### 2.2. Micronutrients Analyses

Copper concentrations in serum, milk, and liver were determined by atomic absorption spectrometry (324 nm) using a method adapted from Dawson et al. [23], as previously described by Galiot et al. [20]. Retinol and 25-OH-D_3_ were measured simultaneously in serum and liver samples using a high-performance liquid chromatographic method adapted from Horst et al. [24] for serum and Jensen et al. [25] for liver. For milk, vitamin D, 24(R),25-dihydroxyvitamin D_3_ and total 25-hydroxyvitamin D (sum of 25-hydroxyvitamin D_3_ and 25-hydroxyvitamin D_2_) were determined by DSM Nutritional Products R&D Analytics (Kaiseraugst, Switzerland), as previously described by Galiot et al. [21]. All of those assays were carried out in duplicate. Unfortunately, retinol could not be determined in milk samples due to uncontrolled technical reasons and a subsequent lack of sufficient sample volumes for repeating another set of analytical procedures.

### 2.3. Oxidative Damage and Antioxidant Enzymes Analyses

Glutathione peroxidase (GPx) and total superoxide dismutase (SOD) activities in plasma, liver, and jejunum samples as well as plasma concentrations of 8-hydroxy-20-deoxyguanosine (8-OHdG) and hepatic ATP were measured as previously described by Galiot et al. [21].

### 2.4. Microbiota Analyses: DNA Isolation, PCR Amplification, Sequencing, and Bioinformatics

The microbial DNA was extracted from colon samples collected from all LW and HW piglets sacrificed at 16, 23, and 42 days of age using the purification Zymo Research Fecal DNA kit (Cedarlane, Burlington, ON, Canada), following the manufacturer’s instructions. Analyses were performed on peri-weaning samples (16 and 23 days of age), only taking into account technical and resource constraints. The final DNA quantity was determined by a PicoGreen-based method (Invitrogen, Burlington, ON, Canada).

The V3–V4 hypervariable regions of the bacterial 16S rRNA gene were amplified using primers 16-F343-Illu and 16S-R803-Illu2 [26] and followed the method as previously performed [20]. Paired-end sequencing (2 × 300 bp) was performed on an Illumina MiSeq system (Genome Québec, Montréal, QC, Canada).

Quality of the readings was assessed using FastQC v.0.11.5 (Babraham Bioinformatics, Cambridge, UK). Analyses were performed within R environment following the DADA2 pipeline. lllumina adapter sequences were trimmed from the raw fastq files using Cutadapt v.1.18, and reads below a window quality score of 20 were trimmed. Reads shorter than 20 bp were discarded, and primers were trimmed in the paired-end mode. Taxonomy assignment was performed with DADA2 against the SILVA reference database (v.132).

### 2.5. Short-Chain Fatty Acids Analyses

Concentrations of SCFAs were determined in caecal digesta using a Perkin Elmer gas chromatograph model Clarus^®^ 580 (Perkin Elmer, Waltham, MA, USA), mounted with a DB-FFAP high-resolution column. Before SCFA quantification, samples were conditioned according to the procedures described by Massé et al. [27].

### 2.6. Statistical Analyses

Data not related to the microbiome were analysed in a split-plot design using the MIXED procedure of SAS (SAS Institute Inc., Cary, NC, USA) in a 2 × 4 factorial arrangement of treatments with supplementation of sows (SUPPL and CONT) and supplementations of piglets (CON, ADCU, BC, and ADCU + BC) as the main factors. Within each litter, the two classes of piglet birth weight (LW vs. HW) were included in the analysis of data as subplots. For repeated measures, the stage of gestation or lactation (sows) or age (piglets) was added to the statistical model. For discrete measurements of treatments administered to piglets, a pairwise comparison test (Tukey–Kramer) was used. For growth performance monitoring and micronutrient status, the group of 4 MW piglets was considered as the experimental unit, whereas for measurements on piglets at slaughter (LW and HW at 16, 23 and 42 days of age), one piglet was considered representative of their experimental unit (LW or HW weight class) at each of these ages.

For microbiome analysis, alpha and beta diversity were computed using R software (version 3.6.0). A principal coordinate analysis (PCoA) and graphical outputs were represented via R software (version 3.6.0) with the package FactoMineR (v.1.42). Phyloseq (v.1.26.1) was used to visualize abundance of microbial taxonomic composition, estimate biodiversity, create heatmaps, and perform normalization and differential abundance tests. Species richness and evenness (Shannon and Simpson index) were calculated for alpha diversity estimations. To compare alpha diversity metrics and relative abundance among groups, a non-parametric Kruskal–Wallis test was conducted, followed by subsequent pairwise comparisons with Benjamini–Hochberg correction. Continuous variables were tested for normality with the Shapiro–Wilk test.

The effects of the statistical model were considered significant at *p* ≤ 0.05 and trends at 0.05 < *p* ≤ 0.10.

## 3. Results

### 3.1. Growth Performance Monitoring and Micronutrient Status of Sows

The mean number ± standard error of the mean (SEM) of piglets born (17.5 ± 0.6) and born alive (15.5 ± 0.9) per litter, as well as the mean sow body weights (±SEM) at 89 and 110 days of gestation (284.8 ± 1.9 and 307.9 ± 2.4 kg, respectively) and one day after weaning (254.9 ± 1.7 kg), did not differ (*p* > 0.10) between sows treatments. The growth performance of piglets could not be reliably assessed because of the different procedures on LW and HW piglets throughout the experimental period. However, values were monitored for all birth weight classes according to ages and are presented in Supplementary Tables S2 and S3.

Serum retinol and Cu concentrations of sows varied from 89 days of gestation to 1 day after weaning (time effect, *p* < 0.01, Table 1), but no effect of sow treatment was observed during the experimental period. For serum 25-OH-D_3_, concentrations increased during the experimental period with values almost three times higher in SUPPL than in CONT sows at weaning (sow treatment × time interaction, *p* < 0.01, Table 1).

In milk collected at 14 days of lactation, copper concentrations were higher in SUPPL (1.19 µg/mL) than in CONT sows (1.06 µg/mL) (*p* < 0.05, SEM = 0.04). Similarly, the total amount of vitamin D tended to be higher in milk of SUPPL sows (2.02 ng/mL) compared with CONT (1.5 ng/mL, *p* < 0.10, SEM = 0.21).

### 3.2. Micronutrient Status in Piglets

Across treatments, concentrations of Cu in the serum of piglets increased during lactation and decreased thereafter from weaning to 42 days of age (age effect, *p* < 0.01, Table 2), but there were no piglet and sow treatment effects during the experimental period. For hepatic Cu, values were higher in ADCU and ADCU + BC compared with CON and BC litters for both 16 and 23 days of age, but this difference disappeared at 42 days of age following a drop of concentrations by approximately 75% in piglets for ADCU and ADCU + BC groups (piglet supplementation × age interaction, *p* < 0.01, Table 3). This post-weaning depletion effect tended to be more pronounced in piglets from supplemented sows (sow supplementation × age interaction, *p* < 0.06, Table 3). Across all ages, values were greater in LW (83.3 µg/g) than in HW piglets (78.8 µg/g) (birth weight class effect, *p* < 0.05, SEM = 4.7).

For retinol, serum concentrations increased from 2 to 8 days of age, then decreased until weaning and increased again at 42 days of age (age effect, *p* < 0.01; Table 2), but there were no piglet and sow treatment effects during the experimental period. In liver, retinol concentrations were greater in SUPPL than CONT litters (*p* < 0.03, Table 3), and this effect was persistent throughout the experimental period. Liver retinol concentrations were also greater in ADCU and ADCU + BC litters (*p* < 0.01) at all ages and decreased after weaning, whereas they increased in BC and CON piglets (piglet supplementation × age interaction, *p* < 0.01, Table 3). LW had higher hepatic retinol level than HW, but only at 16 days of age (293.9 vs. 236.4 µg/g, respectively) (birth weight class × age interaction, *p* < 0.01, SEM= 11.8).

Serum concentrations of 25-OH-D_3_ also increased with the age of piglets (*p* < 0.01). In CON piglets, serum concentrations of 25-OH-D_3_ were considerably lower at 8 and 21 days of age, whereas values were maximized at 8 days of age in treatments ADCU and ADCU + BC and decreased thereafter to levels that did not differ among groups at 42 days of age (piglet supplementation × age interaction, *p* < 0.01, Table 2). There was no sow treatment effect or interaction with piglet treatments on serum concentrations of 25-OH-D_3_ in piglets.

### 3.3. Oxidative Damage and Antioxidant Enzymes Analyses

None of the oxidative stress indicators measured in the plasma (GPx, SOD, and 8-OHdG), liver, and jejunum (cellular and mitochondrial GPx and SOD) were influenced by sow or piglet treatments. Some age and birth weight class effects were observed and are summarized in Table 4. However, hepatic ATP used as a marker of cellular energy status was affected by piglet treatments only at 16 days of age (piglet treatment × age interaction, *p* < 0.02, SEM = 0.80) with lower values in ADCU + BC piglets as compared with the CON (9.89 vs. 14.48 nmol/g, respectively), whereas those for ADCU and BC piglets were intermediate (11.14 and 13.23 nmol/g, respectively.

### 3.4. Microbial Diversity and Relative Abundance of Bacterial Populations in Colon

**Figure 1:**
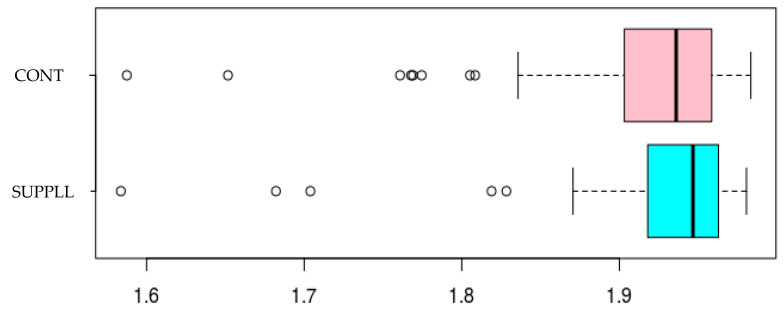
Gut microbial alpha diversity in piglets (average of values across 16 and 23 days of age) as determined by richness rarefied by 2. For sow treatments, gestation and lactation diets were supplemented (SUPPL, *n* = 14 sows) or not (CONT, *n* = 13 sows) with 25-hydroxy-cholecalciferol, β-carotene, and Cu proteinate from 90 days of gestation to weaning at 21 days of lactation. Sow treatment effect, *p* < 0.01.

**Figure 2:**
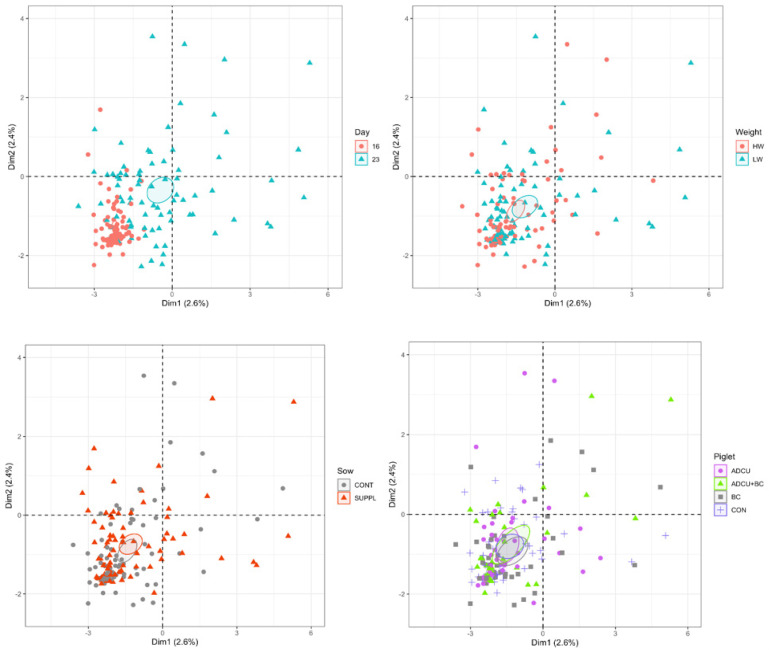
Principal component analysis (PCoA) performed at the operational taxonomic unit level and based on Euclidian distance for all samples. See the text description in Section 3.4 for more details on treatments, birth weight classes, and age responses. For sow treatments, gestation and lactation diets were supplemented (SUPPL, *n* = 14) or not (CONT, *n* = 13) with 25-hydroxy-cholecalciferol, β-carotene, and Cu proteinate from 90 days of gestation to weaning at 21 days of lactation. For piglet treatments, litters were assigned, within each sow treatment, to one of the following treatments: (CON) basal interventions; (ADCU) CON + oral administration of retinol acetate at 2 (4 mg) and 8 d (8 mg) of age, 25-hydroxy-cholecalciferol (100 and 200 µg, respectively), and Cu proteinate (4 and 8 mg, respectively) with an exposure to UVB lights (15 min per day every second day from 5 days of age to weaning); (BC) CON + oral administration of a bovine colostrum extract (4) from 5 to 10 days of age; (ADCU + BC). For birth weight class, LW and HW corresponded to low and high birth weight, respectively, and measurements were performed at 16 and 23 days of age.

The diversity of bacterial populations in piglets was greater at 16 days than at 23 days of age (Shannon index: age effect, *p* < 0.02). The piglets from SUPPL sows had an increased richness (alpha diversity) in bacteria species either before (D16) or after weaning (D23) (sow treatment effect, *p* < 0.01, Figure 1). Piglet supplementation or birth weight class did not have any effect on richness and uniformity before or after weaning. For microbial community structure assessed through a PCoA analysis, no effects of sow’s supplementation, piglet supplementation, or birth weight of piglets (LW and HW piglets) were observed (Figure 2). A shift was observed according to age as the community of bacteria was grouped around a small area from suckling pig at 16 days of age, whereas the overall community distribution was heterogenous in weaned piglets at 23 days of age.

**Figure 3:**
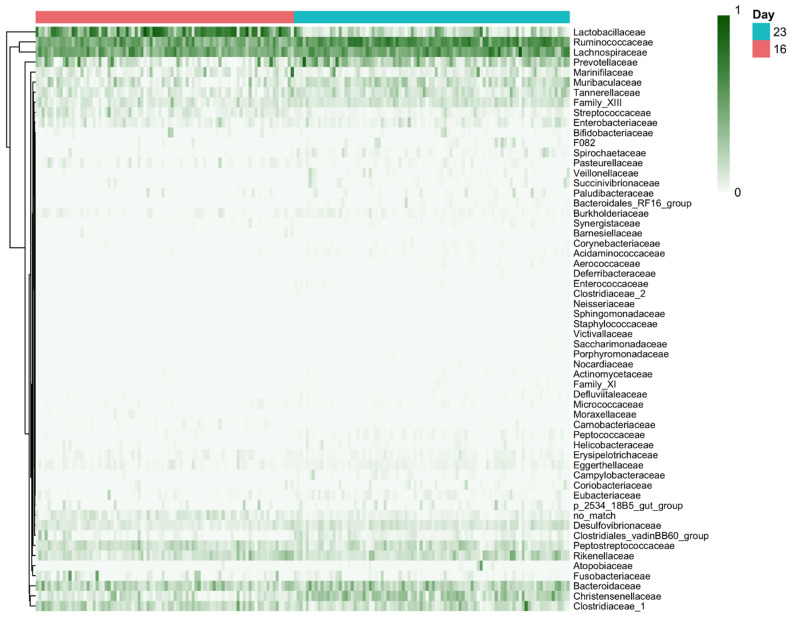
Composition of bacterial communities: Heatmap representing the relative abundance of bacterial families. Families are grouped according to age at sampling, 16 or 23 days. See Section 3.4 for a detailed description of treatments, birth weight classes, and age effects along with their related *p*-values.

In terms of the relative abundance of bacterial populations, the microbial communities of all samples were analysed at the phylum, family, genus, and species taxonomic levels. Firmicutes was the dominant phylum within all piglet microbiota before (16 days of age) and after weaning (23 days of age). For family, the four main abundant groups were *Clostridiaceae* (28%), *Lactobacillaceae* (25%), *Lachnospiraceae* (22.5%), and *Ruminococcaceae* (6.5%) in piglet microbiota for both ages (Figure 3). Treatment responses at the family, genus, and species levels are described in the following text of this section.

For family level, piglets from SUPPL sows had lower relative abundance of *Lactobacillaceae* (CONT: 30% vs. SUPPL: 22%; *p* < 0.05) both before (16 days of age) and after weaning (23 days of age). For abundance of *Bacteroidaceae* in piglet microbiota, values were 13% for piglets from SUPPL sows compared with 11% for CONT sows, but this occurred only after weaning (sow treatment × age interaction, *p* < 0.006). For the *Rykenellaceae* family, piglet treatment effects were only observed within CONT sows, and this was particularly marked for BC piglets after weaning (sow treatment × piglet treatment × age interaction, *p* < 0.01). For the relative abundance of the *Clostridiaceae_1* family, it tended to be higher across ages in LW than in HW piglets (14% vs. 5%, respectively; *p* < 0.08).

For genus level, the *Rikenellaceae_RC9*_gut_group was increased for HW piglets (4.7%) compared with LW piglets (2.8%; *p* < 0.03), although no birth weight class effect was seen at the family level (above). Also, for the genus *Christensenellaceae_R7*_group, there was a higher abundance of this genus in post-weaning (23 days of age) LW piglets receiving BC supplemented treatments (8.1%) vs. their LW littermates unsupplemented with BC (6%) (piglet treatment × birth weight class × age interaction, *p* < 0.03). For *Lactobacillus delbrueckii* species, a quadruple interaction of sow treatment × piglet treatment × age × birth weight class was detected (*p* < 0.01). In such a case, a further separate analysis revealed that the relative abundance of *Lactobacillus. delbrueckii* before weaning at 16 days of age was increased only in LW piglets from SUPPL litters and in those receiving BC as a piglet treatment.

### 3.5. Short-Chain Fatty Acids in Caecum

Effects of sow and piglet treatments were detected on caecal SCFA concentrations of LW and HW piglets, only at 16 days of age (Table 5). Acetate, butyrate, propionate, and valerate concentrations were greater in SUPPL than CONT sows for LW piglets, whereas it was the opposite for HW piglets (sow treatment × birth weight class interaction, *p* < 0.05, Table 5). For piglet treatments, concentrations of acetate, butyrate, and propionate were generally smaller for LW than for HW piglets, and this was more marked for the BC treatment (piglet supplementation × birth weight class interaction, *p* < 0.05, Table 5). For valerate, the birth weight class effect was detected for all supplemented piglets but not in CON (piglet supplementation × birth weight class interaction, *p* < 0.05, Table 5).

## 4. Discussion

### 4.1. Micronutrient Statuses in Sows and Piglets

No effect of sow treatment was observed on Cu serum concentrations of sows during the experimental period. However, the supplementation of Cu to sows did increase by 15% Cu concentrations in milk. This difference is not reflected in serum Cu concentrations in piglets. For piglet supplementation, serum Cu concentrations were not impacted either. This lack of sow or piglet treatment responses on serum Cu concentrations in sows or in piglets was not surprising as serum is not considered a major storage pool for Cu [28] except in situations of insufficient dietary provisions of Cu where serum values may decline [29].

For hepatic Cu concentrations in piglets, values were greater in piglets from SUPPL sows at 16 and 23 days of age, but this effect was no longer apparent after weaning at 42 days of age. For piglet supplementations, responses were substantial with increases of 58% at 16 and 40% at 23 days of age in ADCU and ADCU + BC treatments as compared with the two other piglet treatments. Nevertheless, as for the sow supplementations, such effects have disappeared at 42 days of age. It is noteworthy to mention that values at this age across the different treatments represented a considerable drop in hepatic copper of at least 70% between 21 and 42 days of age. The biological significance of such a drastic decline for the whole-body Cu metabolism remains to be assessed, but it appears that values at that age were minimal at the lifetime scale of these piglets. Actually, the present hepatic Cu concentrations at 42 days of age were much smaller than those measured at birth before the initial ingestion of the dam’s colostrum (average value (± SEM) from 25 individuals of 48.7 ± 2.4 mg/g, Matte et al., unpublished data). In addition, this huge and sudden decline in hepatic Cu concentrations between 21 and 42 days of age occurred in spite of abundant provisions of dietary Cu at 128 (analytical, 124) and 133 (analytical, 111) mg/kg in post-weaning diets given from 21 to 28 and 28 to 42 days of age, respectively. Such high levels of dietary copper, much greater than the NRC [30] recommendation of 6 mg/kg, were used for their bacteriostatic properties along with pharmacological levels of zinc in post-weaning diets at 2729 (analytical, 2343) and 317 (analytical, 340) mg/kg from 21 to 28 and 28 to 42 days of age, respectively. A possible explanation to this apparent huge depletion of copper reserves in liver might be related to the presence of these high concentrations of zinc in the post-weaning diets, as demonstrated by Dalto et al. [31]. In fact, high levels of dietary zinc have been suspected to induce a reduction in copper bioavailability in pigs [32,33].

For vitamin D, supplementation to sows increased their serum concentrations of 25-OH-D_3_ at the end of lactation, and this was in line with a tendency for a 30% increase in vitamin D concentrations in milk at 14 days in lactation. This is in agreement with the transfer of that vitamin through milk observed by Flohr et al. [34], Thayer et al. [35], and Zhang et al. [36]. However, this response in milk was not reflected in serum 25-OH-D_3_ concentrations of piglets during the experimental period, where no effect of sow supplementation was observed. Thayer et al. [35] observed an increase from 4.7 to 7.6 ng/mL in piglets from their corresponding groups of sows receiving supplements similar to those in the present study. However, in the present experiment, as the sow supplementation statistical effect included all piglet treatments, it cannot be excluded that the fourfold increases of 25-OH-D_3_ concentrations in ADCU and ADCU + BC piglets as compared with CON and BC piglets could have interfered with an effect of sow supplementation on 25-OH-D_3_ status in suckling piglets, in spite of the factorial arrangement of sow and piglet treatments. In fact, the most noticeable impact on vitamin D status in suckling piglets was observed with piglet treatments. This was particularly marked when oral supplementations of 25-OH-D_3_ were provided along with UVB throughout the lactation. It is noteworthy to mention that concentrations in these supplemented piglets were transiently higher or equivalent to those of their mother at 8 days of lactation, a response similar to that reported by Jang et al. [37], though with a unique oral supplement of 40,000 IU of vitamin D at one day of age. After weaning, vitamin D was incorporated in the post-weaning diet (1500 IU per kg) for all piglets, and at 42 days of age, no residual effect of treatments persisted. This suggested that the accumulation of 25-OH-D_3_ during early lactation in ADCU and ADCU + BC treatments was not durable during the post-weaning period. This was consistent with the recognized half-life of circulating 25-OH-D_3_ at 15 to 45 days [38] and the fact that no 25-OH-D3 was accumulating in tissue reserves like the liver in piglets [21].

For vitamin A, supplementation of sows increased hepatic concentrations of retinol in piglets from SUPPL sows. This is in line with Heying et al. [39], who reported that pro-vitamin A carotenoid supplementation during gestation and lactation increased both milk concentrations of retinol in sows and liver retinol concentrations of suckling piglets. Out of the three studied micronutrients in this trial, liver vitamin A was the only one where the residual effect of piglet and sow treatments were observed up to 42 days of age. Liver is a well-recognized reserve of vitamin A that is more reactive to variations of dietary vitamin A in piglets than blood serum, which is considered as a transitory pool between reserves and the site of metabolic action [40]. Hepatic concentrations between 200 and 300 µg/g has been considered as a good status of vitamin A in suckling piglets [41]. Such values were not reached in the present experiment at 16 and 23 days of age in piglets without micronutrients supplementation, but in ADCU and ADCU + BC litters, liver retinol concentrations were largely over this threshold, at values between 250 and 400 µg/g. These results were in line with those reported by Surles et al. [40] and Sheftel et al. [42], who showed similar levels of hepatic retinol in suckling piglets after administrations of oral doses between 25 and 50 ĸIU of retinyl acetate or retinyl palmitate in early lactation. Therefore, supplementation in early lactation durably improved the vitamin A status of piglets, at least up to 3 weeks after weaning.

### 4.2. Oxidative Stress and Cellular Energy Metabolism

Copper and vitamins A and D are known to act on some aspects of antioxidant status [38]. However, in spite of the above-described responses in the statuses of these micronutrients, none of the sow or piglet dietary treatments had an impact on markers of antioxidant status in plasma, liver, or jejunum except for hepatic ATP, where this indicator of cellular energy status was minimized in the liver by the combination of piglet treatments (ADCU + BC), which was only at 16 days of age. Such an isolated piglet treatment effect appeared to be tricky to interpret as it is not corroborated by the other different indicators linked to oxidative stress and cellular energy metabolism and was measured at different ages of piglets in the present study. It cannot be excluded that it corresponded to a fortuitous effect without biological significance. Nevertheless, some effects related to age and bodyweight classes were observed (Table 4). These two factors were extensively studied by Novais et al. [17], who showed that (1) weaning was a perturbator of mitochondrial energy production, inducing a durable oxidative stress until at least 35 days of age; and (2) these deleterious effects were more pronounced in low birth weight piglets. Taken together, the present responses were in accordance with Novais et al. [17].

### 4.3. Microbiota in Colon and Short-Chain Fatty Acids in Caecum

The first week post-weaning in piglets is recognized as a period of instability associated with a decreasing diversity of intestinal microbiota [12,43]. This was illustrated in the present study by the smaller Shannon index (indicator of evenness and richness among the microbial population) observed right after weaning. This decrease reflected physiological changes caused by the weaning process with the drastic difference of diets (from sow milk to a solid- and plant-based diet) and with the inflammation that it induces [6]. The present supplementation given to sows during late gestation and lactation increased the richness (alpha diversity) in the colon microbiota of piglets at the end of lactation, and this effect persisted even after weaning. Such a result was in line with the results reported by Zhang et al. [36] following supplementation of 25-hydroxycholecalciferol to sows during a period similar to the present one. In fact, high diversity is usually considered as an indicator of mature gut microbiota and is characterized by an enhanced stability towards environmental threats [44]. Such a maternal response could be explained by the fact that newborn piglets could acquire their intestinal microbiota through their mother via different routes of microbial contacts, such as faeces and vaginal passage at birth [45,46], teat skin [47], and milk [48], as seen for humans during the lactation, as well as the ambient environment [49].

Regarding the overall composition determined by the PCoA analysis, the microbiome composition at the phylum rank showed an overall dominance of Firmicutes for all days followed by Bacteroidetes as previously shown by Schokker et al. [50]. However, no significant effect of any types of supplementation was seen in this experiment. Such results indicated that inter-individual variability of the subset partition of bacterial species among piglets was greater than the effect of sow or piglet supplementations. This was in line with Franzosa et al. [51], who found that the uniqueness in composition of the gut microbiome can be actually used to identify individuals. The present lack of effects of piglet treatments on the diversity or overall composition of microbiota agreed with other studies on copper supplementation to piglets before and after weaning [52] and on vitamin D supplementation [53] in humans (obese or healthy) or on the bovine colostrum as compared with sow milk [10].

Regarding other traits of microbiota, the relative abundance of different taxonomic levels and the products of bacterial fermentation were measured during this experiment, along with short-chain fatty acids (SCFAs, mainly acetate, butyrate, and propionate). These metabolites are considered as products of microbial degradation of carbohydrates and proteins, respectively [54]. They can provide energy to the animal, regulate immune cell development, and suppress inflammation [44,55]. In the present experiment, sow supplementation did increase concentrations of SCFAs in piglets, but this was more marked for LW piglets. This might be associated with the fact that the relative abundance of family *Bacteroidaceae* was greater in SUPPL than in CONT sows as this family is known to produce propionic acid [56] and is capable of using different substrates to produce several SCFAs [57]. However, in spite of these sow’s treatment effects in favor of LW piglets, there were globally more caecal SCFAs in HW piglets. In these piglets, there was a higher abundance of bacteria from the *Rykecenellaceae_RC9*_group. This genus is known to have the metabolic capacity to breakdown proteins and carbohydrates producing several SCFAs [58]. Hence, the higher abundance of this family could contribute to the greater content of SCFAs in HW piglets, providing them with more intestinal endogenous energy than for their LW littermates during lactation. In this way, it has been shown that, during the suckling period, the establishment of an intestinal microbiota including more beneficial bacteria in HW than in LW piglets could be the foundation for the better growth, development, and health in the former group [9]. In relation to piglet treatments, a single effect of BC (the only one in the present experiment) was observed on SCFA concentrations for HW piglets, but this apparent beneficial response could not be associated with any peculiar microbiota profile in these piglets. Generally, it seems that HW piglets have relatively a better capacity to use these extra provisions of nutrients. Inversely, in LW piglets from the BC treatment, the relative abundance of *L. delbrueckii*, a species of bacteria capable of using carbohydrates from milk as a substrate to produce SCFAs [59], was higher at 16 days of age than for their HW littermates, but this was not reflected in concentrations of SCFAs. As mentioned above, several families of bacteria can share the metabolic capacity of producing SCFAs, and it cannot be ruled out that this might have masked the eventual effects of a particular family.

## 5. Conclusions

The strategies used in the present experiment to provide supplemental micronutrients to suckling piglets (indirectly through their dam or directly during lactation with a bovine colostrum extract) were efficient in improving the metabolic status of the different micronutrients (copper and vitamins A and D), but this effect was not generally resilient over the post-weaning period. There was no major impact of supplementations to sows or to piglets on the antioxidative status or acute and cellular energy metabolism of piglets in response to weaning, regardless of their birth weight class. Sow supplementation increased richness of the piglet microbiota in the colon, whereas for SCFAs in the caecum, values were lower in LW piglets than in HW piglets during the suckling period; however, this effect was attenuated for supplemented sows in late lactation. Taken together, the present results indicated that the stress of weaning was a crucial factor over treatment effects or birth weight class effects for the metabolic status of copper and vitamins A and D and for several variables related to control of the antioxidative status and global composition of the microbiota in piglets. Further studies will be needed to investigate the impact of peri-weaning supplementations in these micronutrients, closer to the onset of weaning stress, on these different variables.

## Figures and Tables

**Table 1:** Serum concentrations of retinol, 25-hydroxy-calciferol, and copper in sows during the experimental period.

	Sow Treatments		
Micronutrient/Stage of Gestation–Lactation ^1^	CONT ^2^	SUPPL ^2^	SEM ^3^
Copper, µg/mL ^4^			
89 days of gestation	2.31	2.29	0.03
110 days of gestation	2.24	2.24	0.03
One day after weaning	2.26	2.31	0.04
Retinol, ng/mL ^5^			
89 days of gestation	195.2	198.7	5.6
110 days of gestation	222.7	231.2	9.6
One day after weaning	308.4	273.8	11.2
25-hydroxy-cholecalciferol, ng/mL ^6^			
89 days of gestation	23.9	24.5	0.7
110 days of gestation	23.9	55.7	1.0
One day after weaning	32.3	95.6	2.4

^1^ Weaning at 21 days of lactation. ^2^ Gestation and lactation diets were supplemented (SUPPL, *n* = 24) or not (CONT, *n* = 23) with 25-hydroxy-cholecalciferol, β-carotene, and Cu proteinate from 90 days of gestation to weaning at 21 days of lactation. ^3^ Standard error of the mean. ^4^ Time effect, *p* < 0.03. ^5^ Time effect, *p* < 0.01. ^6^ Sow supplementation × Time interaction, *p* < 0.01.

**Table 2:** Serum micronutrient concentrations of medium-birth weight piglets at 2, 8, 21, and 42 days of age ^1,2^.

	N	Age of Piglets (Days)				SEM ^3^
		2	8	21	42	
Retinol, ng/mL ^4^						
	47	108.7	217.0	196.2	318.1	8.7
Copper, µg/mL ^4^						
	47	0.85	1.98	2.29	1.84	0.04
Piglet treatment	25-hydroxy-cholecalciferol, ng/mL ^5^					
CON	12	0.6	2.0	6.0	21.8	0.9
ADCU	12	0.7	45.9	22.3	22.4	0.9
BC	12	0.9	2.6	5.0	24.0	0.9
ADCU + BC	11	0.9	48.8	21.8	23.2	1.0

^1^ Gestation and lactation diets were supplemented (SUPPL, *n* = 24) or not (CONT, *n* = 23) with 25-hydroxy-cholecalciferol, β-carotene, and Cu proteinate from 90 days of gestation to weaning at 21 days of lactation. ^2^ For each group, litters were assigned to one of the following treatments: (CON) basal interventions; (ADCU) CON + oral administration of retinol acetate at 2 (4 mg) and 8 d (8 mg) of age, 25-hydroxy-cholecalciferol (100 and 200 µg, respectively), and Cu proteinate (4 and 8 mg, respectively) with an exposure to UVB lights (15 min per day every second day from 5 days of age to weaning); (BC) CON + oral administration of a bovine colostrum extract (4 g) from 5 to 10 days of age; and (ADCU + BC). ^3^ Standard error of the mean. ^4^ Age effect, *p* < 0.01. ^5^ Piglet supplementation × age interaction, *p* < 0.01.

**Table 3:** Copper and retinol concentrations (µg/g) in the liver of piglets (average of low and high birth weight) at 16, 23, and 42 days of age.

			Age of Piglets (Days)			SEM ^1^
**Micronutrient**	**Sow Treatment ^2,4^**	**N**	**16**	**23**	**42**	
Copper, µg/g	CONT	23	95.9	108.4	30.6	3.9
	SUPPL	24	106.3	120.1	25.2	3.7
Retinol, µg/g	CONT	23	250.3	240.1	223.0	7.5
	SUPPL	24	280.0	270.7	233.7	7.5
**Micronutrient**	**Piglet Treatment ^3,5^**					
Copper, µg/g	CON	12	75.5	94.2	25.1	5.3
	ADCU	12	123.3	127.9	28.5	5.3
	BC	12	82.4	96.4	22.4	5.3
	ADCU + BC	11	123.2	138.5	35.6	5.6
Retinol, µg/g	CON	12	144.5	161.0	193.7	8.3
	ADCU	12	371.7	348.9	249.1	11.8
	BC	12	146.3	166.2	202.0	8.3
	ADCU + BC	11	398.6	345.4	268.5	12.5

^1^ Standard error of the mean. ^2^ Gestation and lactation diets were supplemented (SUPPL, *n* = 14) or not (CONT, *n* = 13) with 25-hydroxy-cholecalciferol, β-carotene, and Cu proteinate from 90 days of gestation to weaning at 21 days of lactation. ^3^ For each group, litters were assigned to one of the following treatments: (CON) basal interventions; (ADCU) CON + oral administration of retinol acetate at 2 (4 mg) and 8 d (8 mg) of age, 25-hydroxy-cholecalciferol (100 and 200 µg, respectively), and Cu proteinate (4 and 8 mg, respectively) with an exposure to UVB lights (15 min per day every second day from 5 days of age to weaning); (BC) CON + oral administration of a bovine colostrum extract (4) from 5 to 10 days of age; (ADCU + BC). ^4^ Sow supplementation × age interaction, *p* < 0.06 for copper and *p* < 0.03 for retinol. ^5^ Piglet supplementation × age interaction, *p* < 0.01 for both micronutrients.

**Table 4:** Plasma antioxidant enzyme activities and oxidant injury products, hepatic cellular energy status, and hepatic and jejunum antioxidant enzyme activities according to age and birth weight class of piglet.

	Blood Plasma					
	Age of Piglets (Days)			Birth Weight Class ^1^		SEM ^2^
	16	23	42	LW	HW	
GPx, mU/mg ^3^	13.7	17.7	-	16.2	15.2	0.5
SOD plasma, U/mg ^4^	0.15	0.14	-	0.17	0.12	0.01
8-OHdG, pg/mL ^3^	7.6	6.6	-	7.2	7.0	0.2
Liver						
ATP, nmol/g ^5^	12.2	9.5	12.8	12.2	10.7	0.8
Mitochondrial SOD, U/mg ^6^	5.6	7.8	6.0	6.3	6.6	0.3
SOD total, U/mg ^5^	20.2	24.5	23.7	22.2	23.4	0.8
Mitochondrial GPx, mU/mg ^6^	104.3	107.8	132.5	115.1	114.6	5.2
GPx total, mU/mg ^6^	214.3	202.7	266.5	227.5	228.1	7.3
Jejunum						
Mitochondrial SOD, U/mg ^6^	3.1	3.2	2.8	3.1	3.0	0.2
SOD total, U/mg	6.5	6.9	5.2	6.2	6.3	0.2
GPx total, mU/mg ^6^	32.4	34.8	42.1	36.3	36.5	2.1

^1^ LW: Low body weight; HW: High body weight. ^2^ Standard error of the mean. ^3^ Age effect, *p* < 0.001, weight class effect, *p* < 0.02. ^4^ Weight class effect, *p* < 0.001. ^5^ Age effect, *p* < 0.001, weight class effect, *p* <0.05. ^6^ Age effect, *p* < 0.001.

**Table 5:** Concentrations of short-chain fatty acids in mg/L of caecal digesta collected at 16 days of age in low and high birth weight piglets according to sow and piglet treatments.

	Weight Class of Piglets		
	HW ^1^	LW ^1^	SEM ^2^
**Sow treatment ^3,4^**			
CONT			
Acetate	2702	2142	226
Butyrate	529	320	60
Propionate	1092	823	89
Valerate	229	148	30
SUPPL			
Acetate	2303	2285	221
Butyrate	470	348	58
Propionate	997	982	87
Valerate	225	169	29
**Piglet treatments ^5,6^**			
CON			
Acetate	2195	2227	245
Butyrate	304	301	53
Propionate	909	852	91
Valerate ^7^	159	158	37
ADCU			
Acetate	2525	2381	311
Butyrate	582	441	104
Propionate	1044	1028	140
Valerate ^7^	284	122	52
BC			
Acetate	2805	2046	368
Butyrate	658	275	88
Propionate	1167	808	149
Valerate ^7^	284	122	36
ADCU + BC			
Acetate	2480	2236	299
Butyrate	460	324	61
Propionate	1071	935	98
Valerate ^7^	220	161	29

^1^ LW: Low birth weight; HW: High birth weight. ^2^ Standard error of the mean. ^3^ Gestation and lactation diets were supplemented (SUPPL, *n* = 14) or not (CONT, *n* = 13) with 25-hydroxy-cholecalciferol, β-carotene, and Cu proteinate from 90 days of gestation to weaning at 21 days of lactation. ^4^ Sow treatment × birth weight class interaction, *p* < 0.05 for all short-chain fatty acids. ^5^ For each group, litters were assigned to one of the following treatments: (CON) basal interventions; (ADCU) CON + oral administration of retinol acetate at 2 (4 mg) and 8 d (8 mg) of age, 25-hydroxy-cholecalciferol (100 and 200 µg, respectively), and Cu proteinate (4 and 8 mg, respectively) with an exposure to UVB lights (15 min per day every second day from 5 days of age to weaning); (BC) CON + oral administration of a bovine colostrum extract (4) from 5 to 10 days of age; (ADCU + BC). ^6^ Piglet supplementation × birth weight class interaction, *p* < 0.05. ^7^ Piglet supplementation × birth weight class interaction, *p* < 0.05.

## Data Availability

The data presented in this study are available on request from the corresponding author.

## Supplementary Materials

The following supporting information can be downloaded at: https://www.mdpi.com/article/10.3390/vetsci12020151/s1, Table S1: Composition of sow’s diets during lactation and gestation; Table S2. Body weight of low birth weight and high birth weight piglets during lactation and after weaning; Table S3. Growth performance of medium-birth weight piglets during lactation and after weaning.
