# Supplementary material for: Effect of Neonatal Interventions with Specific Micronutrients and Bovine Colostrum on Micronutrient and Oxidative Statuses and on Gut Microbiota in Piglets from Birth to Post-Weaning Period
Source: Vet Sci. 2025 Feb 10;12(2):151. doi: 10.3390/vetsci12020151 (PMC11860533; doi:10.3390/vetsci12020151)
Supplement: Supplementary file 1 [file vetsci-12-00151-s001.zip › vetsci-3408117-supplementary.pdf]

Table S1. Composition of sow's diets during lactation and gestation.

	Gestation	Lactation
**Ingredients, %**		
Maize	59.1	65.1
Middling wheat	18.8	-
Soybean hulls	10.0	-
Distillers dried grain with solubles	7.9	-
Soybean meal (48% PB)	-	23.1
Canola meal	1.1	6.0
Limestone	1.4	1.9
Salt	0.55	0.48
Monocalcium phosphate	0.39	1.2
Fat	-	1.5
Mineral[1] and vitamins[2] Premix	0.30	0.30
L-Lysine	0.25	0.20
Choline Chloride	0.08	0.08
Anti-mold	0.05	0.05
L-Thréonine	0.08	0.05
Méthionine	-	0.04
**Analytical nutrients composition**		
ME, MJ/kg	12.6	13.3
CP, %	13.3	17.6
CF, %	6.3	3.0
Ca, %	0.79	1.2
P, %	0.56	0.61
K, %	0.73	0.86
Mg, %	0.23	0.19
Na, %	0.26	0.25
Fat, %	3.0	3.5
Ash, %	4.8	6.1
Vitamin A, UI/kg	11,129	9,023
Vitamine D_3, UI/kg	1,935	1,593

Copper, µg/g	33.6	15.8

[1]Provided per kg of diet: manganese, 37 mg; zinc, 143 mg; copper, 16 mg; iron, 125 mg; iode, 2.05 mg; selenium, 303 µg.
[2] Provided per kg of diet: Vitamin A, 9,984 UI; Vitamin D_3, 1,440 UI; Vitamin E, 60 UI; Vitamin K (menadion), 2.5 mg; Vitamin B_1 (thiamin), 2,0 mg; Vitamin B_2 (riboflavin), 6,0 mg ; Vitamin B_3 (niacin), 40 mg; Vitamin B_5 (pantothenic), 20 mg; folic acid, 7.5 mg ; Vitamin B_6 (pyridoxine), 2.5 mg ; Vitamin B_8 (biotine), 400 µg ; vitamin B12, 20 µg.

Table S2. Body weight of low birth weight and high birth weight piglets during lactation and after weaning.

**Birth weight class**	**High birth weight**	**SEM**	**Low birth weight**	**SEM**
Body weight, 1 day of age, kg[1]	1.8	0.2	1.2	0.2
Body weight, 21 days of age (weaning), kg[2]	8.3	0.9	6.6	1.2
Body weight, 28 days of age, kg[3]	9.4	1.0	7.9	1.2
Body weight, 42 days of age, kg[4]	17.0	2.0	14.7	2.1

[1] Average of 4 piglets per birth weight class within each litter.
[2] One piglet was sacrificed at 16 days of age.
[3] One piglet was sacrificed at 23 days of age.
[4] One piglet was sacrificed at 42 days of age.

Table S3. Growth performance of medium birth weight piglets during lactation and after weaning.

	Treatments to sows		
**Lactation period**	**CONT[1]**	**SUPPL [1]**	**SEM[2]**
Body weight, 1 day of age, kg[3]	1.46	1.50	0.02
Body weight, 21 days of age, (weaning), kg[3]	7.68	7.40	0.10
Body weight, 28 days of age, kg[3]	8.78	8.68	0.12
Body weight, 42 days of age, kg[3]	15.6	16.2	0.2
Average daily gain, 0-21 days of age, g/day[4]	311	295	6
Average daily gain, 21-28 days of age, g/day	157	184	12
Average daily gain, 28-42 days of age, g/day[5]	488	540	18
Feed intake, 21-28 days of age, g/day	185	205	13
Feed intake, 28-42 days of age, g/day	636	672	18
Gain:Feed ratio 21-28 days of age, g/kg	850	897	29
Gain:Feed ratio 28-42 days of age, g/kg	782	802	13

[1]Gestation and lactation diets were supplemented (SUPPL, n=24) or not (CONT, n=22) with 25-hydroxy-cholecalciferol, β-carotene et Cu-proteinate from 90 days of gestation to weaning at 21 days of lactation.
[2]Standard error of the mean.
[3]Sow treatment x Age interaction, $P < 0.01$.
[4]Sow treatment effect, $P < 0.06$.
[5]Sow treatment effect, $P < 0.08$.
